# *Coriolus versicolor* Mushroom Grown on Selenium-Rich Zeolitic Tuff as a Potential Novel Food Supplement

**DOI:** 10.17113/ftb.60.01.22.7172

**Published:** 2022-03

**Authors:** Danka Matijašević, Milena Pantić, Nemanja Stanisavljević, Sanja Jevtić, Nevenka Rajić, Steva Lević, Viktor Nedović, Miomir Nikšić

**Affiliations:** 1 University of Belgrade, Faculty of Agriculture, Nemanjina 6, 11080 Belgrade-Zemun, Serbia; 2 University of Belgrade, Institute of Molecular Genetics and Genetic Engineering, Vojvode Stepe 444a, 11042 Belgrade, Serbia; 3 University of Belgrade, Faculty of Technology and Metallurgy, Karnegijeva 4, 11000 Belgrade, Serbia

**Keywords:** *Coriolus versicolor*, mushrooms, Se-rich zeolitic tuff, antioxidant enzymes, biological potential

## Abstract

**Research background:**

In the recent years, considerable attention has been given to selenium status since its deficiency is linked with various disorders and affects at least 13% of world population. Additionally, mushrooms are known to possess pronounced capacity for absorption of various micronutrients, including Se, from soil/substrate. Here, we investigate the possibility of using Se-rich zeolitic tuff as a supplement for production of selenized mushroom. Furthermore, the impact of the enrichment on the activity of antioxidant enzymes and biological potential of *Coriolus versicolor* medicinal mushroom is studied.

**Experimental approach:**

Se(IV)- and Se(VI)-modified natural zeolitic tuff from the Serbian deposit Zlatokop was used as supplement for mushroom cultivation. To examine the effectiveness of selenium enrichment, we determined total selenium with inductively coupled plasma mass spectrometry (ICP-MS), together with the activity of antioxidant enzymes in fresh fruiting bodies and biological potential of methanolic extracts. Antioxidant activity was evaluated using the appropriate tests for: inhibition of lipid peroxidation, DPPH free radical scavenging assay, Fe(III)-reducing antioxidant power assay and ability of chelating Fe^2+^ ions. The antibacterial activity against foodborne pathogens was measured by broth microdilution assay. Additionally, chemical composition of the prepared extracts was studied using UV-Vis and Fourier transform infrared (FTIR) spectroscopy.

**Results and conclusions:**

Content of selenium detected in biofortified *C. versicolor* was even 470 times higher than in control on dry mass basis ((140.7±3.8) *vs* (0.3±0.1) µg/g), proving that Se-rich zeolitic tuff is an excellent supplement for mushroom production. Furthermore, the results of monitoring the activity of antioxidant enzymes revealed that most of the Se-enriched mushrooms exhibited higher superoxide dismutase (SOD) and catalase (CAT) and lower glutathione peroxidase (GSH-Px) activities than control. Due to higher amounts of enzymes, which can quickly catalyze the reduction of superoxide radicals, the quality of selenium-enriched mushrooms is preserved for a longer period of time. Investigation of biological potential indicated that Se-enriched mushroom methanolic extracts, generally, expressed enhanced antioxidant properties. Additionally, extracts showed antibacterial activity against all tested pathogenic microorganisms.

**Novelty and scientific contribution:**

Cultivation of mushrooms on Se-enriched zeolitic tuff is a new technological approach for obtaining Se-fortified food/supplements with enhanced antioxidant and antibacterial activities.

## INTRODUCTION

For the past few decades, selenium has been the subject of extensive research due to its fundamental importance to human health (*1*-*3*). As selenocysteine, selenium is a constituent of selenoproteins, some of which have important antioxidant roles. One of the best known and explored families of selenium-dependent enzymes are glutathione peroxidases, which protect membrane lipids and phospholipids from oxidative stress (*1*). Along with highly specialized enzymes such as SOD and CAT, GSH-Px represents the first line of defense against free radical attack. Low or diminishing selenium status is linked with various disorders such as reduced immune function, increased susceptibility to viral infection, thyroid dysfunction, increased cancer and cardiovascular disease risk, oxidative stress-related conditions, *etc*. (*1*, *3*). Its deficiency affects at least 13% of world population, mostly European countries and some parts of Asia, specifically China, Tibet and Siberia, where daily selenium intake is approx. 7–30 µg/day (*2*, *3*). Current recommendations for intake of Se referred as the reference nutrient intake (RNI) range between 70 and 55 µg/day for adult males and females, respectively (*4*). Since the meeting of daily Se requirements in humans would result in improved public health and cost savings, different solutions for increasing Se in regions where this element is in short supply in the food chain are needed.

As nontoxic, inexpensive and available materials, zeolites have been used as a carrier for fertilizers in plant production, notably due to their ability to slowly release nutrients (*5*). Primary building units of zeolites are SiO_4_/AlO_4_ tetrahedra organized in the form of three-dimensional structure with cavities or channels, which provide high porosity. Their main characteristics are ion exchange ability, catalytic activity and adsorption (*6*). Previous research showed that the addition of zeolite to the substrate may accelerate the growth of mushroom mycelia, shorten the time required for the formation of fruiting bodies and improve their chemical composition (*7*).

Apart from their exquisite taste and flavour, for which they have been known for centuries, mushrooms also synthesize a large number of diverse bioactive compounds with potent pharmacological properties (*8*). To date, a number of papers relating to the antibacterial, antioxidant, immunomodulating, antidiabetic, hypolipidaemic and anticancer properties of numerous mushroom extracts have been published (*9*-*11*). Several categories of bioactive components, including polysaccharides (mainly glucans, *e.g.* β-1,6 and β-1,3), peptides and proteins, phenolics, flavonoids, antioxidant enzymes, as well as minerals such as selenium and zinc are present in mushrooms (*12*-*15*). Previously published research showed that mushrooms, cultivated on a substrate supplemented with Se, can accumulate this micronutrient in high concentrations (*3*, *15*). Additionally, Se-enrichment improved mushroom shelf-life (*2*), had an impact on total phenolic and antioxidant properties (*15*) and provided a good source of bioavailable Se (*3*). *Coriolus versicolor* (*Trametes versicolor* (L.:Fr.) Lloyd, 1920), white-rot (saprophytic) fungus is a medicinal mushroom with a wide array of physiological activities that has been used, specifically in China, as a tonic and drug for thousands of years (*9*). Numerous studies revealed that *C. versicolor* extracts possess significant immunomodulatory or immunostimulatory effects, as well as strong antioxidant, antitumour, antimicrobial, hepatoprotective and analgesic activities (*9*, *10*, *16*). If *C. versicolor* can successfully accumulate Se from substrate/soil, then it could be explored as a great potential for correcting deficiency of this trace element and possibly have additional health benefits provided by the specific properties of mushroom.

The aim of this study is to evaluate the possibility of use of the Se-enriched zeolite as a supplement for mushroom production. Furthermore, the impact of Se enrichment on the activity of antioxidant enzymes in fresh fruiting bodies of *C. versicolor* was examined. To our knowledge, the information about their activity in mushrooms is scarce. Finally, *in vitro* antioxidant and antibacterial activities of the methanolic extracts of Se-enriched mushroom were investigated. To explain the results, chemical composition of the prepared extracts was established using inductively coupled plasma mass spectrometry (ICP-MS), UV-Vis and FTIR spectroscopy analyses.

## MATERIALS AND METHODS

### Preparation of Se(IV)- and Se(VI)-modified zeolite

Modification of zeolitic tuff (CLI; which originated from Zlatokop deposit in Vranjska Banja, Serbia) and adsorption of selenite and selenate ions were conducted according to the method of Jevtić *et al.* (*17*). Briefly, as a carrier for Se(IV) and Se(VI) oxoions, a previously prepared Fe(III)-loaded clinoptilolite (Fe-CLI) was used. Selenium oxoions were adsorbed by suspending the Fe-CLI into the solution of Na_2_SeO_3_ (4.5·10^–3^ mol/dm^3^; Sigma-Aldrich, Merck, St. Louis, MO, USA) or Na_2_SeO_4_ (3.0·10^–3^ mol/dm^3^; Sigma-Aldrich, Merck) and mixing suspensions for 24 h. The solid/liquid ratio was 1:100 and the pH was adjusted to 8 or 3 for selenite and selenate, respectively. The suspensions were separated, the solids were dried at 105 °C overnight and the obtained products were denoted Se(IV)-CLI or Se(VI)-CLI.

### Growth of Coriolus versicolor on media with Se-rich zeolitic tuff

Pure culture of basidiomycete, *Coriolus versicolor* (which originated from the Košutnjak, park-forest near Belgrade, Republic of Serbia), was obtained from the collection of the Department of Industrial Microbiology, Faculty of Agriculture, University of Belgrade. The substrate consisted of a mixture of oak sawdust, wheat straw and wheat bran (in ratio 5:3:2). Substrates without (control sample) or with Se-rich zeolitic tuff in the mass fraction of 50 mg/kg (Se(IV)-CLI 50 or Se(VI)-CLI 50) or 62.5 mg/kg (Se(IV)-CLI 62.5 or Se(VI)-CLI 62.5) were used for experiments. Upon sterilization, 10% inocula, prepared as previously described (*17*), were added to each bag (Microsac, Sac O_2_, Deinze, Belgium). During 20 days mycelia had completely colonized the substrate, after which fructification occurred at (20±2) °C with 80–95% relative humidity (growth chamber GC-1000TLH; Jeio Tech, Daejeon, Korea). The mushrooms were harvested and used for determination of antioxidant enzymes or air dried at 40 °C to a constant mass and ground to fine powder (*16*, *17*).

### Determination of antioxidant enzymes in C. versicolor fresh fruiting bodies

#### Protein isolation

The fresh fruiting bodies of Se-enriched mushrooms and control sample were weighed (3 g), cut into small pieces and then thoroughly ground in a cold mortar with pestle using liquid nitrogen. Prepared samples were extracted (3 mL/g fresh mass) in 50 mM phosphate buffer (pH=7.8; Sigma-Aldrich, Merck) and 0.1 mM EDTA (Calbiochem, San Diego, CA, USA), sonicated 3 times for 20 s at a frequency of 10 kHz and then centrifuged (centrifuge AG 5804R; Eppendorf, Hamburg, Germany) for 20 min at 2350×*g* and 4 °C (*12*). In the obtained supernatants, the total protein content was established according to Bradford method (*18*) using bovine serum albumin as a standard (Carl Roth, GmBH & Co.KG, Karlsruhe, Germany). Moreover, the activities of antioxidant enzymes (superoxide dismutase (SOD), glutathione peroxidase (GSH-Px) and catalase (CAT)) were also determined. Additionally, screening of the protein profile using sodium dodecyl sulfate polyacrylamide gel electrophoresis (SDS-PAGE) was performed.

#### Superoxide dismutase activity assay

The total superoxide dismutase (SOD) activity was determined by the method outlined by Beauchamp and Fridovich (*19*). Briefly, the reaction mixture consisted of 50 mM phosphate buffer (pH=7.8), 0.1 mM EDTA, 13 mM l-methionine (Merck Millipore, Burlington, VT, USA), 75 µM nitro blue tetrazolium chloride (NBT) (SERVA Electrophoresis GmbH, Heidelberg, Germany), 2 µM riboflavin (Supelco, Bellefonte, PA, USA) and an appropriate aliquot of the extract. Riboflavin was added last and the reaction was initiated by placing the tubes under the fluorescent light (30 W) for 10 min at room temperature. Absorbance was read at 560 nm using an Ultrospec 3300 pro UV/Vis spectrophotometer (Amersham Biosciences, Amersham, UK). One unit of the SOD activity was defined as the amount of enzyme required for 50% inhibition of NBT reduction and expressed in unit per milligram of protein.

#### Glutathione peroxidase activity assay

The activity of glutathione peroxidase (GSH-Px) (E.C. 1.11.1.9) was determined using Ransel kit (Randox Laboratories Ltd., Crumlin, UK). GSH-Px catalyzes the oxidation of glutathione (GSH) to glutathione disulfide (GSSG) by organic peroxides (cumene hydroperoxide). In the presence of glutathione reductase and NADPH the oxidized GSSG is instantly converted to the reduced form (GSH) with an accompanying oxidation of NADPH to NADP^+^. The decrease in absorbance was measured at 340 nm (Ultrospec 3300 pro UV/Vis spectrophotometer; Amersham, Biosciences). One unit of the GSH-Px activity was defined as the amount of enzyme that oxidized 1 µmol of NADPH per min and expressed in unit per gram of protein. The activity of GSH-Px was calculated using the following equation:

GSH-Px activity=[((Δ*A*_s_-Δ*A*_b_)·*V*_rm_)/(*ε*·*γ*_p_·*V*_s_)] /1/

where Δ*A*_s_ is the average change in the absorbance of the sample per minute, Δ*A*_b_ is the average change in the absorbance of the blank per minute, *V*_rm_ is the volume of the reaction mixture (mL), *γ*_p_ is protein concentration in the sample (mg/mL), *V*_s_ is the sample volume (mL), and *ε* is the molar absorption coefficient of NADH at 340 nm (6220 L/(mol·cm)).

#### Catalase activity assay

The catalase (CAT) activity was determined according to the method described by Claiborne (*20*). The activity of the enzyme was quantified by the decomposition of H_2_O_2_ (Merck Millipore) into H_2_O and O_2_ at 240 nm (Ultrospec 3300 pro UV/Vis spectrophotometer, Amersham). One unit of the CAT activity was defined as the amount of enzyme that reduced 1 µmol of H_2_O_2_ per min and expressed in unit per milligram of protein. The activity of catalase was calculated using the following equation:

CAT activity=[((Δ*A*_s_-Δ*A*_b_)·*V*_rm_)/(*ε*·*γ*_p_·*V*_s_)] /2/

where Δ*A*_s_ is the average change in the absorbance of the sample per minute, Δ*A*_b_ is the average change in the absorbance of the blank per minute, *V*_rm_ is the volume of the reaction mixture (mL), *γ*_p_ is protein concentration in the sample (mg/mL), *V*_s_ is the sample volume (mL), and *ε* is the molar absorption coefficient of H_2_O_2_ at 240 nm (43.6 L/(mol·cm)).

#### Sodium dodecyl sulfate-polyacrylamide gel electrophoresis

Sodium dodecyl sulfate-polyacrylamide gel electrophoresis (SDS-PAGE) was performed according to the procedure previously described by Laemmli (*21*). Briefly, 7.5 μL protein samples (each containing 15 µg protein) were mixed with 2.5 μL sample loading buffer, heated at 90 °C for 5 min and then loaded into 12% acrylamide gel, 1.0 mm thickness (SERVA Electrophoresis GmbH) together with the pre-stained SDS-PAGE standard 26616 Page Ruler^TM^, 10−to 180 kDa (Thermo Fisher Scientific, Waltham, UK).

### Preparation of C. versicolor methanolic extracts

Mushroom methanolic extracts were prepared as previously described by Matijašević *et al.* (*16*). The dried mushroom samples (10 g) with 150 mL methanol (LGC Promochem, Wesel, Germany) were extracted by stirring at 120 rpm for 24 h (room temperature), followed by filtration through a Whatman No. 4 paper. The residues were additionally re-extracted twice under the same conditions. Methanol was removed from the combined extracts using a rotary evaporator type R-II (Buchi, Flawil, Switzerland) at 40 °C to dryness.

### Determination of total selenium in fruiting bodies and methanolic extracts

Approximately 0.25 g (the exact mass was measured to four decimals) of dried and thoroughly chopped samples of fruiting bodies or methanolic extracts were taken for digestion in 5 mL HNO_3_ (Merck KGaA, Darmstadt, Germany) at 260 °C in closed-vessel microwave-assisted system (Milestone, Bergamo, Italy). After sample preparation, inductively coupled plasma mass spectrometry (ICP-MS, Agilent 8800 QQQ ICP-MS; Agilent Technologies, Inc., Santa Clara, CA, USA) equipped with MassHunter software was applied for determination of total selenium (*22*). For the accuracy tests, a certified soil reference material: bush, branches and leaves (NCS DC 73348) from the China National Analysis Center for Iron and Steel (Beijing, PR China) were used.

### Qualitative chemical analysis of methanolic extracts

The chemical composition of methanolic extracts of *C. versicolor* was analyzed using attenuated total reflection Fourier transform infrared (ATR-FTIR) spectrometer IRAffinity-1 (Shimadzu, Kyoto, Japan). All measurements were performed in the spectral range 4000–600 cm^–1^ with a resolution of 4 cm^–1^ (*16*).

### Quantitative chemical analysis of methanolic extracts

The total carbohydrates were determined according to the method described by Sawangwan *et al.* (*23*) and d-glucose (Sigma-Aldrich, Merck) was used to produce the standard calibration curve. Briefly, each extract (0.25 mg/mL) was mixed with phenol (5%) and sulfuric acid (96%) after which the absorbance was measured at 490 nm. The yeast and mushroom β-glucan assay kit (Megazyme Int., Wicklow, Ireland) was used for the measurement of the contents of total and α-glucans, according to manufacturer’s instructions. Total proteins were determined by the method outlined by Bradford (*18*) using bovine serum albumin as the standard reference. The content of total soluble phenolics in the extracts was measured using Folin–Ciocalteu reagent (Merck KGaA) and 6% Na_2_CO_3_ (Sigma-Aldrich, Merck) (*13*), while the total flavonoids were determined according to the assay described by Sknepnek *et al.* (*24*). Gallic acid (Merck KGaA) and (+)-catechin (Sigma-Aldrich, Merck) were used as standards for determination of total soluble phenolics and total flavonoids, respectively. All analyses were performed with the UV-1800 spectrophotometer (Shimadzu).

### Evaluation of antioxidant properties of the extracts

The antioxidant activity was determined by the conjugated diene method in linoleic acid model system (*13*). Different concentrations of extracts were mixed with 10 mM linoleate emulsion (prepared by mixing linoleic acid (Fluka Chemie GmbH, Buchs, Switzerland), methanol, 0.2 M sodium phosphate buffer (pH=6.5) and 6.5 mM Tween 20 (Sigma-Aldrich, Merck) and tested for antioxidant activity at 234 nm using the spectrophotometer model UV-1800 (Shimadzu). Ascorbic acid (Merck KGaA) and α-tocopherol (Merck KGaA) served as positive controls.

DPPH free radical scavenging activity was measured according to the method described by Klaus *et al.* (*11*). In the first series each extract was mixed with dimethyl sulfoxide (DMSO) solution (Sigma-Aldrich, Merck) of 0.2 mM DPPH (Sigma-Aldrich, Merck), while in the second series extracts were mixed only with DMSO solution. The radical scavenging activity was monitored at 517 nm (spectrophotometer UV-1800; Shimadzu) and calculated as a percentage of DPPH discolouration. Ascorbic acid, butylated hydroxytoluene (BHT; Sigma-Aldrich, Merck) and α-tocopherol were used as positive controls.

The Fe(III)-reducing antioxidant power (FRAP) assay was determined according to Kozarski *et al.* (*13*). Shortly, each extract in methanol was mixed with phosphate buffer (pH=6.6), 1% K_3_Fe(CN)_6_ (Sigma-Aldrich, Merck), 10% trichloroacetic acid (TCA; Sigma-Aldrich, Merck), Milli-Q water and 0.1% FeCl_3_ (Sigma-Aldrich, Merck). The absorbance was measured at 700 nm (spectrophotometer UV-1800; Shimadzu), while ascorbic acid was used for comparison.

Chelating ability of Fe(II) ions was determined using the method by Miletić *et al.* (*4*). Briefly, the reaction mixture consisted of methanol, 2 mM FeCl_2_ (Sigma-Aldrich, Merck), 5 mM ferrozine (Sigma-Aldrich, Merck) and an appropriate aliquot of the extract dissolved in methanol. The ability of each extract to chelate Fe(II) ions was measured at 562 nm (spectrophotometer UV-1800; Shimadzu). Citric acid (Sigma-Aldrich, Merck) and EDTA (Merck KGaA) were used as positive controls.

Applied concentrations of mushroom methanolic extracts ranged from 0.1 to 2.5 mg/mL in DPPH free radical scavenging activity assay, from 0.1 to 10 mg/mL in the antioxidant activity assay and from 0.1 to 20 mg/mL in chelating ability and FRAP assays.

The EC_50_ values were also calculated, representing the effective concentrations of the extracts at which 50% of the antioxidant activity was reached.

### Antibacterial activity assay

#### Bacterial strains and culture preparation

Five Gram-positive (*Listeria monocytogenes* ATCC 19111, *Staphylococcus aureus* ATCC 25923, *Staphylococcus epidermidis* ATCC 12228, *Bacillus cereus* ATCC 11778 and *Enterococcus faecalis* ATCC 29212) and five Gram-negative bacterial species (*Pseudomonas aeruginosa* ATCC 27853, *Shigella sonnei* ATCC 29930, *Yersinia enterocolitica* ATCC 27729, *Salmonella* ser. Enteritidis ATCC 13076 and *Escherichia coli* O157:H7 ATCC 35150) were used for the antibacterial activity testing. All bacterial strains utilized in this study originated from American Type Culture Collection (ATCC, Manassas, VA, USA). Tryptic soy broth/agar (HiMedia Laboratories, Mumbai, India) was used for *L. monocytogenes* and *E. coli* O157:H7 culture preparation, while for all the other strains Müeller Hinton broth/agar (HiMedia Laboratories) was used. The final concentration of bacterial suspensions was adjusted to approx. 10^5^–10^6^ CFU/mL (*16*).

#### Broth microdilution assay

Minimum inhibitory (MIC) and minimum bactericidal concentrations (MBC) of *C. versicolor* methanolic extracts were assayed using standard broth microdilution method (*4*, *16*). The concentrations of the mushroom extracts ranged from 40 to 0.3125 mg/mL.

### Statistical analysis

All experiments were conducted in triplicate and the results are expressed as mean value±S.D. The obtained data were subjected to a one-way analysis of variance (ANOVA) and Tukey’s HSD test to identify signiﬁcant diﬀerences (α=0.05) among the means. For the statistical analysis Origin Pro v. 9.0 (*25*) and MS Excel (Microsoft Office 2007 Professional) (*26*) were employed.

## RESULTS AND DISCUSSION

### Se(IV)- and Se(VI)-enriched zeolite

Structural investigation obtained by X-ray powder diffraction (XRPD) showed that the zeolitic tuff consisted mainly of clinoptilolite (CLI) phase (72.6%), while feldspar (14.6%) and quartz (12.8%) were present to a lesser extent (*27*). The results of the adsorption capacity of Se on the Fe-CLI surface showed that its efficiency depends on the oxidation state of selenium: 21 mg/g of SeO_3_^2–^ ions and 17 mg/g of SeO_4_^2–^ ions were adsorbed from the solution.

### Accumulation of Se in C. versicolor

Table 1 shows the results of the accumulation of Se in *C. versicolor*. Se supplementation at both tested mass fractions did not reduce or slow down the growth of the fruiting bodies. Generally, the content of total Se was significantly higher in Se-enriched mushrooms than in the control sample. When 50 mg/kg were added, selenium content in fruiting bodies was approx. 400–470 times higher than in the control sample, while the addition of 62.5 mg/kg caused an increase of 350 times. Previous research (*4*, *28*) established that *Coriolus* species are able to accumulate Se, but in this study it is shown for the first time that Se-enriched zeolite can be an excellent source of Se since detected mass fractions of this element were very high. An explanation for the obtained results possibly lies in the ability of zeolite to act as a pH regulator by binding hydrogen ions. By examining Se uptake by field crops and vegetables, De Temmerman *et al.* (*29*) noticed that soil pH greatly influences the availability of Se, namely at higher pH (neutral and even alkaline soil) the uptake of Se significantly increased. In alkaline soils, Se becomes water soluble and thus available to plants. Furthermore, Jevtić *et al.* (*17*) showed that desorption of the same amount of Se (10 mg) from Se(VI)-CLI was approx. 3 times faster than from Se(IV)-CLI, indicating that Se(VI) ions form a more labile complex with the Fe-CLI. This could be the explanation for the generally better results obtained for Se accumulation in *C. versicolor* grown on the substrate with the addition of Se(VI)-CLI.

**Table 1:** Selenium mass fraction in *Coriolus versicolor* fruiting body and methanolic extracts

*w*(Se)_added_/(mg/kg)	*w*(Se)_total_/(µg/g)		
Se(IV)-CLI		Se(VI)-CLI
Fruiting body			
0		0.3±0.1	
50	(121.0±6.9)^b^		(140.7±3.8)^a^
62.5	(110.8±4.1)^a^		(105.6±3.0)^a^
Methanolic extract			
0		0.47±0.03	
50	(110.1±3.4)^b^		(119.3±2.6)^a^
62.5	(96.0±4.4)^a^		(86.3±4.1)^b^

Data are expressed as mean value±S.D. (*N*=3). Within the same row, mean values followed by different letters are significantly different at α≤0.05 according to ANOVA and Tukey’s HSD test. Se(IV)-CLI and Se(VI)-CLI=mushroom enriched with Se(IV) and Se(VI)-rich zeolitic tuff, respectively

The analysis of total Se content in extracts revealed that most of the Se was extracted with methanol and its mass fraction ranged from approx. 85 to 120 µg/g. According to these findings, it can be concluded that Se is predominantly found in compounds soluble in alcohol.

### The activity of antioxidant enzymes in C. versicolor fresh fruiting bodies

**Fig. 1:**
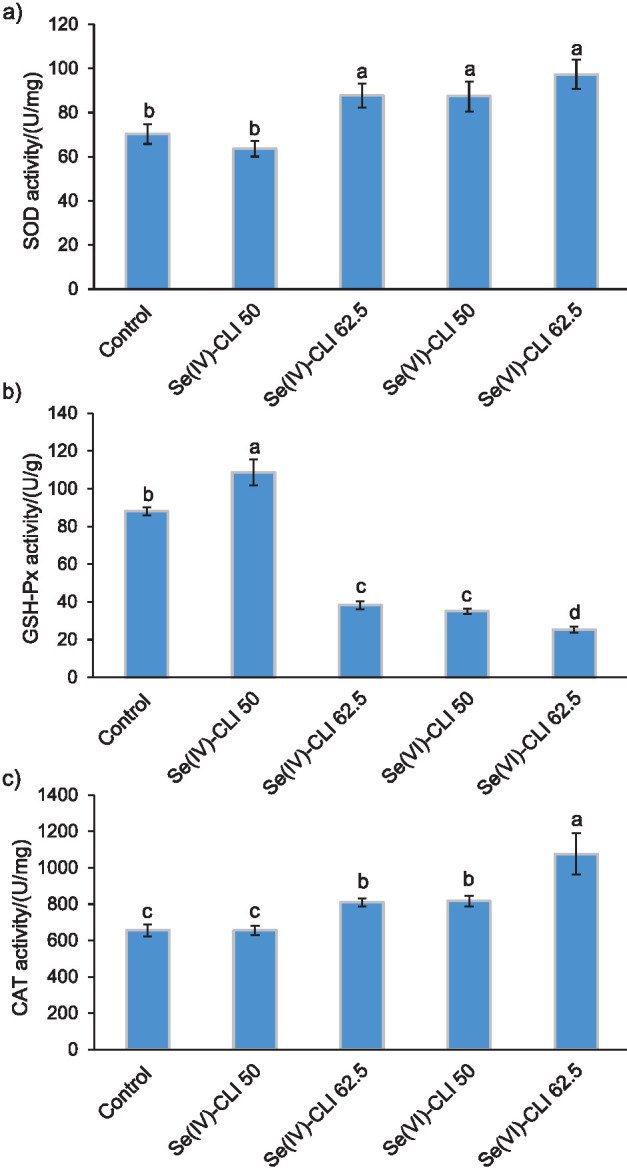
The activities of: a) superoxide dismutase (SOD), b) glutathione peroxidase (GSH-Px) and c) catalase (CAT) in control and Se-enriched *Coriolus versicolor* mushrooms. Each value is expressed as mean±S.D. (*N*=3). Mean values followed by different letters are significantly different at α≤0.05 according to ANOVA and Tukey’s HSD test. Se(IV)-CLI 50 and Se(IV)-CLI 62.5=mushroom enriched with 50 and 62.5 mg/kg Se(IV)-rich zeolitic tuff repectively, Se(VI)-CLI 50 and Se(VI)-CLI 62.5=mushroom enriched with 50 and 62.5 mg/kg Se(VI)-rich zeolitic tuff respectively

Fig. 1 shows the superoxide dismutase (SOD), glutathione peroxidase (GSH-Px) and catalase (CAT) activities of Se-enriched and non-enriched *C. versicolor* mushrooms. With the exception of Se(IV)-CLI 50, Se-enriched mushrooms manifested higher SOD and CAT activities, approx. from 24 to 38% and from 24 to 65%, respectively, than the non-enriched control. On the other hand, sample Se(IV)-CLI 50 had the highest GSH-Px activity ((108.7±7.0) U/g), while the activity of the other Se-enriched samples was in the range from 25 to 40 U/g and was markedly lower than of the control ((88.0±2.2) U/g).

The activity of GSH-Px was reduced in the following order: Se(IV)-CLI 50>control>Se(IV)-CLI 62.5≈Se(VI)-CLI 50>Se(VI)-CLI 62.5. Since GSH-Px shares a common substrate (*i.e.* H_2_O_2_) with CAT, the obtained results could be explained by the CAT activity of the examined samples that decreased in the opposite order. Namely, the samples with the highest CAT activity had the weakest GSH-Px activity, and *vice versa*. GSH-Px has a much higher affinity for H_2_O_2_, and thus acts as the primary defense against a low, continuous exposure to H_2_O_2_, while CAT becomes more important in the conditions of acute, severe oxidative stress, since it cannot be saturated by H_2_O_2_ at any concentration (*30*).

Numerous experiments showed that fungal decomposition is caused by a combination of factors acting on wood polymers, such as production of different radicals (*e.g.* hydroxyl), enzymes (like carbohydrate-active enzymes or oxidative enzymes), metabolites, *etc.* (*31*). The survival of the fungus in such reactive oxygen species (ROS)-rich environment suggests the existence of efficient activation of an intracellular antioxidant defense system, including antioxidant enzymes (*32*). These findings are consistent with the results of Cheng *et al.* (*12*), who reported significantly higher activity of SOD in mushrooms (50–400 U/mg) than in algae, bacteria and plants (between 0.1 and 59 U/mg). Additionally, the activity of CAT in mushrooms was also proved to be high, around 550 U/mg (*33*).

Xiong *et al.* (*34*) showed that transformation of an antioxidant glutathione peroxidase gene restored the ability of mushroom fruiting, while the activity of GSH-Px increased approx. 2.5 times (from 5 to 13 U/g). This research confirmed that the inability of fungal cultures to deal with the accumulated ROS is the most likely cause of their degeneration, which consequently leads to a significant commercial loss.

To our knowledge, there are very few reports on the impact of selenium enrichment on the activity of mushroom antioxidant enzymes. Dong *et al.* (*35*) showed that selenium-enriched *Cordyceps militaris* had higher SOD activity (57.23 U/mg) than the mushroom grown on the substrate without sodium selenite addition (36.35 U/mg) and especially than wild *C. militaris* (10.32 U/mg).

Following harvest, mushroom continues to develop; however, under the conditions of nutritional and water deprivation, a massive disruption in metabolism is induced. Most of these changes lead to a formation of free radicals and ROS, which are counteracted by antioxidant enzymes. Harvested mushrooms that have greatly elevated transcript level of SOD are able to respond quickly to a potentially lethal level of superoxide radicals and thus improve their postharvest quality and tolerance to abiotic stress (*36*). Another study (*37*), which focused on the impact of a high oxygen atmosphere on mushrooms, also showed a connection between higher amounts of SOD, peroxidase and CAT and delays in senescence (postponed occurrence of browning in mushrooms).

### Molecular mass distribution of proteins in Se-enriched C. versicolor

**Fig. 2:**
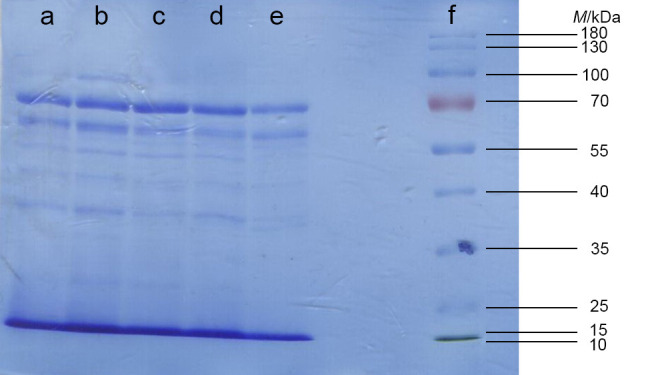
SDS-PAGE analysis of total proteins extracted from *Coriolus versicolor* mushroom. Five sample bands and the standard protein kit of different molecular masses for calibration are visible: a=control (non-enriched) sample, b=Se(IV)-CLI 50 sample, c=Se(IV)-CLI 62.5 sample, d=Se(VI)-CLI 50 sample, e=Se(VI)-CLI 62.5 sample and f=standard protein kit. Se(IV)-CLI 50 and Se(IV)-CLI 62.5=mushroom enriched with 50 and 62.5 mg/kg Se(IV)-rich zeolitic tuff respectively, Se(VI)-CLI 50 and Se(VI)-CLI 62.5=mushroom enriched with 50 and 62.5 mg/kg Se(VI)-rich zeolitic tuff respectively

The results of the SDS-PAGE electrophoresis (Fig. 2) indicated that proteins or their subunits extracted from Se-enriched *C. versicolor* mushrooms had a wide range of molecular masses, from approx. 25 to 100 kDa. Among the about ten detected bands, proteins with molecular masses from 55 to 70 kDa were predominant. It was also observed that selenium did not affect the protein distribution of Se-fortified *C. versicolor* samples, which was previously demonstrated by other authors (*38*).

Previous research showed that Se could be distributed in the proteins with molecular masses ranging from 8.7 to 142.5 kDa (*38*), with the highest amount (around 80%) incorporated into proteins at <36.3 kDa (*39*). Similar results were published by Tie *et al.* (*40*), who found at least six Se-containing proteins in the Se-enriched *Flammulina velutipes* mushroom extract, and their molecular masses ranged from 9 to 74 kDa. In more detailed analysis, Hu *et al.* (*41*) found three water-soluble selenoproteins in *Agaricus blazei* Murrill mushroom. The first selenoprotein was identified as an isoenzyme of isocitrate dehydrogenase with molecular mass of 114 kDa, the second was a kind of dihydrolipoyl dehydrogenase (molecular mass of 54 kDa), while the third was characterized as a kind of d-proline reductase (molecular mass of 47 kDa). Additionally, selenocystine was found to be predominant Se unit in all three selenoproteins.

Furthermore, it has been established that the low content of proteins with molecular mass above 150 kDa is due to physical damage of the cell wall of fungi or the activity of endogenous proteases (*42*). Additionally, low Se mass fractions can stimulate the activities of proteases, which hydrolyse proteins to small peptides and/or amino acids (*39*). According to this, the resulting protein fractions with molecular masses below 100 kDa, obtained in this study, were expected.

### Chemical composition of methanolic extracts

The chemical analyses of the extracts revealed that the amount of examined components depended on the mass fraction of Se added in the substrate (Table 2). When 50 mg/kg were added, higher total polysaccharide (from 13 to 19%), as well as total glucan and β-glucan (17–37%) content were detected than in the previous report on non-enriched (control) *C. versicolor* methanolic extract (*16*). On the other hand, the addition of higher mass fractions of Se (62.5 mg/kg) increased the total phenol (10–15%) and flavonoid contents (approx. 4.5 times). In all methanolic extracts, the main polysaccharide components were β-glucans, indicating that glucans are primarily linked by β-type glycosidic bonds. All Se-enriched extracts had higher content of total proteins (from 23-127%) than control sample (*16*).

**Table 2:** Chemical composition of *Coriolus versicolor* methanolic extracts

Extract	*w*(polysaccharide) _total_/(mg/g)	*w*(glucan)/(mg/g)			*w*(protein)_total_/(mg/g)	*w*(phenolics as GAE)_total_/(mg/g)	*w*(flavonoid as CE)_total_/(mg/g)
Total	α	β
Control	(351.0±19.0)^b^	(203.0±11.0)^c^	(6.0±0.5)^a^	(197.0±9.0)^c^	(8.0±0.5)^d^	(25.8±1.4)^b^	(4.3±0.2)^d^
Se(IV)-CLI 50	(398.3±8.9)^a^	(233.8±10.1)^b^	(3.2±0.6)^b^	(230.6±7.5)^b^	(18.2±1.0)^a^	(25.5±0.3)^b^	(8.2±0.2)^c^
Se(IV)-CLI 62.5	(353.3±7.7)^b^	(204.0±8.5)^c^	(6.6±0.9)^a^	(197.4±8.6)^c^	(14.8±0.4)^b^	(28.6±0.3)^a^	(19.8±0.9)^a^
Se(VI)-CLI 50	(417.6±12.1)^a^	(272.8±11.4)^a^	(2.6±0.4)^b^	(270.2±8.4)^a^	(9.9±0.4)^c^	(24.8±0.6)^b^	(9.0±0.3)^c^
Se(VI)-CLI 62.5	(355.7±7.4)^b^	(204.6±9.2)^c^	(6.7±0.8)^a^	(197.9±7.7)^c^	(14.8±0.6)^b^	(29.7±0.4)^a^	(17.5±0.8)^b^

All results are on a dry mass basis of extract and represent mean value±S.D. (*N*=3). Mean values followed by different letters within the same column are significantly different at α≤0.05 according to ANOVA and Tukey’s HSD test. GAE=gallic acid equivalent, CE=catechin equivalent, Se(IV)-CLI 50 and Se(IV)-CLI 62.5=extract obtained from mushroom grown on 50 and 62.5 mg/kg Se(IV)-rich zeolitic tuff respectively, Se(VI)-CLI 50 and Se(VI)-CLI 62.5=extract obtained from mushroom grown on 50 and 62.5 mg/kg Se(VI)-rich zeolitic tuff respectively

Generally, the information about chemical composition of *Coriolus* extracts, notably Se-enriched one, is limited. Miletić *et al.* (*4*) reported that methanolic extracts of selenium-enriched *C. versicolor* mycelia contained between 22.0 and 23.3 mg/g of total phenolics. The same authors determined significantly lower mass fraction of total glucans and total proteins (6.8–8.3 and 3.8–4.1 mg/g, respectively) than those found in this study, while total polysaccharide mass fraction ranged between 114.1 and 271.0 mg/g.

As previously documented, Se treatment increased the phenolic content of different white rot fungi (*14*, *43*, *44*). Enhanced phenol biosynthesis may be explained as a part of a mechanism of detoxification due to contamination with Se ions or as an indirect effect caused by the inhibition of the enzyme activity of polyphenol oxidase by antioxidant-active selenium compounds (*43*). Moreover, higher mass fractions of phenolics may also be ascribed to the capability of Se to enhance the accumulation of some sugars, *e.g.* glucose, which are an important substrate in many metabolic pathways. The phenolic and flavonoid biosynthesis may also be stimulated under stress conditions such as low temperature, metals, injury, *etc*. (*14*).

### FTIR spectra of the methanolic extracts

**Fig. 3:**
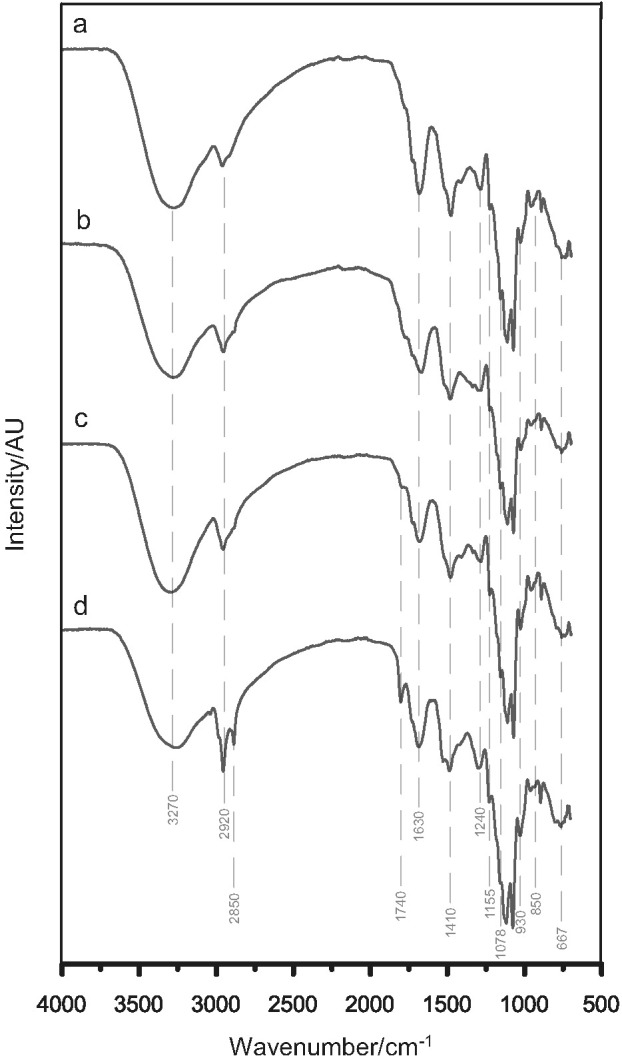
FTIR spectra of Se-enriched *C. versicolor* methanolic extracts: a=Se(IV)-CLI 62.5, b=Se(VI)-CLI 62.5, c=Se(IV)-CLI 50 and d=Se(VI)-CLI 50. Se(IV)-CLI 50 and Se(IV)-CLI 62.5=extract obtained from mushroom grown on 50 and 62.5 mg/kg Se(IV)-rich zeolitic tuff respectively, Se(VI)-CLI 50 and Se(VI)-CLI 62.5=extract obtained from mushroom grown on 50 and 62.5 mg/kg Se(VI)-rich zeolitic tuff respectively

Fig. 3 shows the FTIR spectra of methanolic extracts of Se-enriched *C. versicolor*. We identified the following bands in the spectra of *C. versicolor* methanolic extract related to primary metabolites of mushroom: 3500–3000 cm^–1^ (OH-groups (*13*, *45*) and N–H bonds (*13*)), ~2920 cm^–1^ (CH_2_ stretching vibrations (*45*)), and ~2850 cm^–1^ (CH stretching vibrations (*46*)) was noticed only for Se(VI)-CLI 50 extract. The residual proteins may be identified by bands at ~1410 and ~1240 cm^–1^ (*13*, *45*). The spectra of Se(IV)-CLI 50 and Se(VI)-CLI 50 showed an absorption band at 1750–1690 cm^–1^ due to C=O group (*13*, *45*). The most pronounced spectral differences between control (*16*) and Se-enriched extracts were noticed at ~1630 cm^–1^, which was ascribed to absorption due to carbon-oxygen double-bond asymmetric stretching vibration (*47*). Bands between 1410 and 1310 cm^–1^ (OH groups (*13*)) confirm the presence of the secondary metabolites such as phenolic compounds. The bands at ~1155 and ~1080  cm^–1^ are due to sugar ring in the polysaccharide molecules (*45*). The extracted β-glucans are identified by a shoulder near 890 cm^–1^, while the weak bands at ~930 cm and ~850 cm^–1^ indicated the presence of α-glycosidic bonds (*13*, *45*). A wide band around ~667 cm^–1^, detected in all extracts, was ascribed to the Se–O–C symmetric stretching vibration and indicated that Se was successfully extracted with methanol (*4*). Results obtained by FTIR spectroscopy are in accordance with those obtained by quantitative chemical analysis, and indicated the dominant presence of polysaccharides, while protein, lipids and pigment compounds were also extracted but to a lesser extent.

### Antioxidant potential of C. versicolor methanolic extracts

#### Antioxidant activity determined by the conjugated diene method

**Fig. 4:**
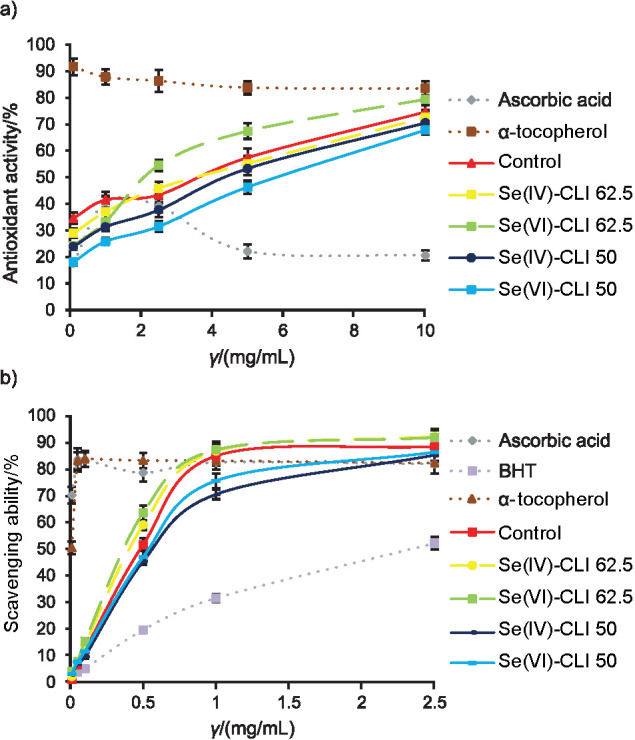
Antioxidant activity evaluated by: a) the conjugated diene method, and b) scavenging ability on DPPH radicals of control and Se-enriched *Coriolus versicolor* methanolic extracts. Se(IV)-CLI 50 and Se(IV)-CLI 62.5=extract obtained from mushroom grown on 50 and 62.5 mg/kg Se(IV)-rich zeolitic tuff respectively, Se(VI)-CLI 50 and Se(VI)-CLI 62.5=extract obtained from mushroom grown on 50 and 62.5 mg/kg Se(VI)-rich zeolitic tuff respectively. Each value is expressed as mean±S.D. (*N*=3)

Using this method, all *C. versicolor* methanolic extracts showed the same patterns, *i.e.* their antioxidant activities increased with the increase in their concentrations (Fig. 4a). Furthermore, it was noticed that the antioxidant activity does not depend on the type of oxoions, but on the concentration of Se added to the substrate. Namely, the lowest EC_50_ values were obtained for samples Se(IV)-CLI 62.5 and Se(VI)-CLI 62.5 ((3.94±0.17) and (2.75±0.09) mg/mL, respectively). Regression analysis found that antioxidant activity strongly correlates with the total phenol content (r=–0.82), which is in agreement with previously published data (*10*, *46*). Additionally, no correlation was established with total glucan content (*11*, *13*).

As previously documented, Se-enriched mushrooms showed excellent results in preventing lipid peroxidation (*44*), and their antioxidant activity was enhanced by 400% compared to the non-enriched sample (*43*). The results point to the important role of Se, particularly sourced from mushrooms, in preventing lipid peroxidation and preserving the integrity and functioning of tissues and cells.

#### DPPH free radical scavenging activity assay

The scavenging ability of control, Se(IV)-CLI 62.5 and Se(VI)-CLI 62.5 extracts steadily increased to a concentration of 1 mg/mL after which a plateau was reached, while the ability of Se(IV)-CLI 50 and Se(VI)-CLI 50 was dose dependent, *i.e.* increased as the concentration increased (Fig. 4b). Significantly higher potential of Se(IV)-CLI 62.5 and Se(VI)-CLI 62.5 extracts towards DPPH radicals was confirmed by their low EC_50_ values ((0.77±0.03) and (0.73±0.01) mg/mL, respectively). Furthermore, the same observation was noted as in the previous assay, *i.e.* the scavenging capacity depended on the concentration of Se added to the substrate, and not on the type of oxoions. Additionally, at the highest tested concentration (2.5 mg/mL), all mushroom extracts were more effective than the used positive controls: ascorbic acid (81.8%), α-tocopherol (82.1%) and especially BHT (52.1%).

Regression analysis revealed very strong correlation between EC_50_ values and total phenol (r=–0.96) and flavonoid content (r=–0.84), while no correlation was established with total polysaccharide content. The obtained results are in agreement with Klaus *et al.* (*11*).

Compared with previously published data, it seems that scavenging ability of control extract was comparable with the activity found in other strains of *C. versicolor* (*10*, *48*). Considering the results for Se-enriched mushrooms, it was established that different *Pleurotus* species showed a significant increase of total phenol content and DPPH inhibition with increasing concentrations of Se added to the substrate (*14*). Moreover, it was documented that Se-enriched extracts showed enhanced scavenging abilities on DPPH, OH**^˙^** and O_2_**^˙^**ˉ radicals compared to the non-enriched extracts (*43*, *44*, *49*).

#### Reducing power

**Fig. 5:**
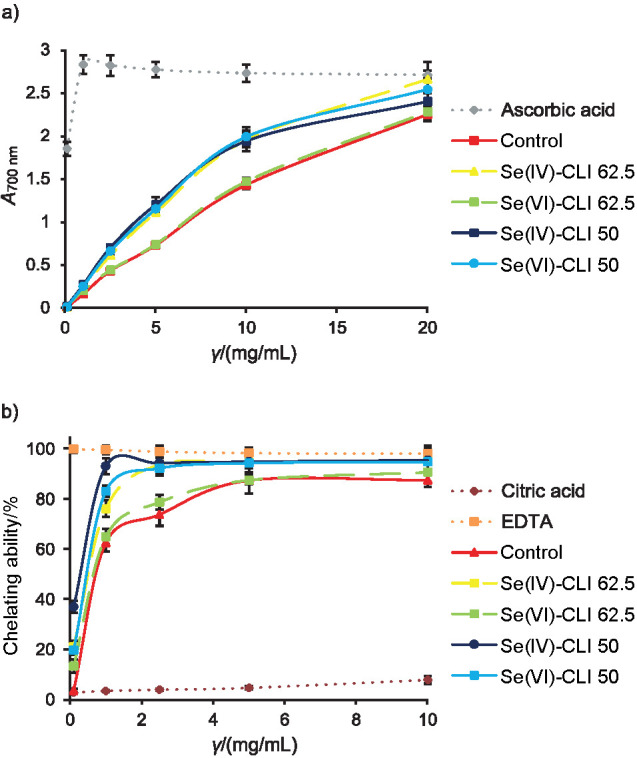
Antioxidant potential determined by: a) reducing power, and b) chelating ability of Fe^2+^ ions of control and Se-enriched *C. versicolor* methanolic extracts. Se(IV)-CLI 50 and Se(IV)-CLI 62.5=extract obtained from mushroom grown on 50 and 62.5 mg/kg Se(IV)-rich zeolitic tuff respectively, Se(VI)-CLI 50 and Se(VI)-CLI 62.5=extract obtained from mushroom grown on 50 and 62.5 mg/kg Se(VI)-rich zeolitic tuff respectively. Each value is expressed as mean±S.D. (*N*=3)

All *C. versicolor* methanolic extracts showed a high potential of hydrogen-donating ability and their reducing activities increased readily along with their increased concentrations (Fig. 5a). At lower concentrations (from 2.5 to 10 mg/mL), the reducing power of Se(IV)-CLI 50, Se(VI)-CLI 50 and Se(IV)-CLI 62.5 extracts was up to 60% higher than of control extract. Furthermore, EC_50_ values of all Se-enriched samples (ranging from (1.9±0.1) to (2.90±0.09) mg/mL) revealed higher potential than that found in control ((3.3±0.1) mg/mL).

Using regression analysis, it was noticed that the correlation between EC_50_ and total polysaccharide content was strong and negative (r=–0.73) as well as the correlation between EC_50_ and β-glucan content (r=–0.65). Negative and moderate correlation between EC_50_ values and total protein content was also established (r=–0.53). These results could be explained by the presence of the reducing amino acids such as methionine and cysteine in the protein components of the mushroom extracts. Correlation between reducing power and selenium content was found to be strong and negative (r=–0.88).

Turło *et al.* (*43*) reported that the reducing power of Se-enriched *Lentinula edodes* mycelial methanolic extract was even 30% higher than of the non-enriched one, which is in accordance with the obtained results. Except hydrogen-donating abilities, stronger reducing power of medicinal mushrooms might also be ascribed to a higher amount of reductones, which react with radicals (*48*). Reductones can form directly from the sugar in uncatalyzed reactions. Lo *et al.* (*50*) reported that hydroxyl groups of polysaccharides, present in extracts, can act as electron donors and thus terminate the radical chain reaction. Moreover, the same authors concluded that the glycosyl linkage of the side chain structure contributed greatly to the reducing reaction.

#### Chelating ability of Fe^2+^ ions

All extracts had the same pattern of chelating ability, *i.e.* at first their binding of Fe^2+^ ions was strongly concentration dependent, after which a plateau was reached and further increase of extract concentration had only slight impact on the chelating (Fig. 5b). All Se-enriched extracts expressed stronger chelating ability (from 90.8 to 95.7%) than control (87.7%), which was also confirmed by their lower EC_50_ values (from (0.30±0.01) to (1.10±0.03) mg/mL; control: (1.32±0.04) mg/mL). Correlation between EC_50_ values and total selenium content was strong (r=–0.82), while it was moderate and negative with total polysaccharide (r=–0.70) and protein content (r=–0.66).

As previously documented, Se-enriched *Ganoderma lucidum* polysaccharide extract exhibited high antioxidant properties, probably as a result of the ability of selenium to complex the free metal ion. Selenium most likely exists in the form of selenyl group (–SeH) or seleno-acid ester in the polysaccharides containing Se (*49*). Hart *et al.* (*51*) showed that selenium decreases radical- and non-radical-induced oxidative DNA damage through formation of metal coordination complexes.

### Antibacterial potential of C. versicolor methanolic extracts

Table 3 shows the results of the antibacterial testing of Se-enriched *C. versicolor* methanolic extracts. Se-enriched extracts exhibited stronger inhibitory and bactericidal effect on Gram-positive than on Gram-negative bacteria. The same results were previously obtained for non-enriched (control) *C. versicolor* methanolic extract (*16*). The most susceptible Gram-positive bacteria were *S. epidermidis* (MIC ranged from (1.25±0.00) to (5.0±0.0) mg/mL) and *B. cereus* (MIC=(5.0±0.0) mg/mL). On the other hand, *L. monocytogenes* is singled out as the most resistant bacteria, since none of the extracts was bactericidal in the tested range of concentrations. Among Gram-negative bacteria, *S. sonnei* was the most sensitive (MIC ranged from (2.5±0.0) to (5.0±0.0) mg/mL), followed by *Y. enterocolitica* (MIC ranged from (5.0±0.0) to (10.0±0.0) mg/mL) and *S.* Enteritidis (MIC=(10.0±0.0) mg/mL). With the exception of Se(VI)-CLI 62.5 extract, which was lethal to four out of five tested Gram-negative bacteria, the MBC values of other extracts were not determined. Additionally, extracts prepared from mushrooms supplemented with higher concentration of Se (Se(IV)-CLI 62.5 and Se(VI)-CLI 62.5) expressed the same or lower MIC and MBC values against tested Gram-negative bacteria than Se(IV)-CLI 50 and Se(VI)-CLI 50 extracts. As can be seen in Table 1, Table 2 and Fig. 3, total Se mass fraction, chemical composition and the structure of polysaccharide components greatly differ among extracts and, therefore, the obtained differences in the antibacterial activity were expected. The mechanisms based on which Se expresses its antibacterial activity are still under question, but it is assumed that ROS are produced upon exposure of the bacterial cultures to Se nanoparticles (*52*). Additionally, Se treatment may increase the phenolic content of extracts (*14*, *43*) and thus indirectly contribute to antibacterial activity since phenols are toxic to microorganisms depending on the site(s) and number of hydroxyl groups (*16*).

**Table 3:** Antibacterial activity of Se-enriched *Coriolus versicolor* methanolic extracts determined by the broth microdilution assay

B Bacterial strain	Assay	*γ*/(mg/mL)			
Se(IV)-CLI 50	Se(IV)-CLI 62.5	Se(VI)-CLI 50	Se(VI)-CLI 62.5
*S. aureus* ATCC 25923	MIC	(10.0±0.0)^a^	(5.0±0.0)^b^	(5.0±0.0)^b^	(2.5±0.0)^c^
	MBC	n.d.	(20.0±0.0)^a^	(10.0±0.0)^b^	(10.0±0.0)^b^
*S. epidermidis* ATCC 12228	MIC	(1.25±0.00)^b^	(5.0±0.0)^a^	(5.0±0.0)^a^	(1.25±0.00)^b^
	MBC	(1.25±0.00)^b^	(10.0±0.0)^a^	(10.0±0.0)^a^	(1.25±0.00)^b^
*E. faecalis* ATCC 29212	MIC	(10.0±0.0)^a^	(10.0±0.0)^a^	(10.0±0.0)^a^	(5.0±0.0)^b^
	MBC	n.d.	(20.0±0.0)^b^	(40.0±0.0)^a^	(40.0±0.0)^a^
*B. cereus* ATCC 11778	MIC	(5.0±0.0)^a^	(5.0±0.0)^a^	(5.0±0.0)^a^	(5.0±0.0)^a^
	MBC	(5.0±0.0)^a^	(5.0±0.0)^a^	(5.0±0.0)^a^	(5.0±0.0)^a^
*L. monocytogenes* ATCC 19111	MIC	(20.0±0.0)^b^	(40.0±0.0)^a^	(20.0±0.0)^b^	(20.0±0.0)^b^
	MBC	n.d.	n.d.	n.d.	n.d.
*E. coli* O157:H7 ATCC 35150	MIC	(40.0±0.0)^a^	(40.0±0.0)^a^	(40.0±0.0)^a^	(40.0±0.0)^a^
	MBC	n.d.	n.d.	n.d.	n.d.
*S.* Enteritidis ATCC 13076	MIC	(10.0±0.0)^a^	(10.0±0.0)^a^	(10.0±0.0)^a^	(10.0±0.0)^a^
	MBC	n.d.	n.d.	n.d.	40.0±0.0
*S. sonnei* ATCC 29930	MIC	(5.0±0.0)^a^	(2.5±0.0)^b^	(5.0±0.0)^a^	(2.5±0.0)^b^
	MBC	n.d.	n.d.	n.d.	40.0±0.0
*Y. enterocolitica* ATCC 27729	MIC	(10.0±0.0)^a^	(5.0±0.0)^b^	(10.0±0.0)^a^	(5.0±0.0)^b^
	MBC	n.d.	n.d.	n.d.	40.0±0.0
*P. aeruginosa* ATCC 27853	MIC	(40.0±0.0)^a^	(40.0±0.0)^a^	n.d.	(40.0±0.0)^a^
	MBC	n.d.	n.d.	n.d.	40.0±0.0

Data are expressed as mean value±S.D. (*N*=3). Mean values followed by different letters within the same row are significantly different at α≤0.05 according to ANOVA and Tukey’s HSD test. n.d.=not determined (with the highest tested concentration of samples (40 mg/mL), antibacterial activity was not determined). Se(IV)-CLI 50 and Se(IV)-CLI 62.5=extract obtained from mushroom grown on 50 and 62.5 mg/kg Se(IV)-rich zeolitic tuff respectively, Se(VI)-CLI 50 and Se(VI)-CLI 62.5=extract obtained from mushroom grown on 50 and 62.5 mg/kg Se(VI)-rich zeolitic tuff respectively

## CONCLUSIONS

In conclusion, the results of the study demonstrated that Se-containing zeolitic tuff can be successfully used as a supplement for the production of Se-enriched mushrooms. Selenium mass fraction in fresh fruiting bodies ranged from (105.6±3.0) to (140.7±3.8) µg/g, and in methanolic extracts between (86.3±4.1) and (119.3±2.6) µg/g. Analysis of the activity of antioxidant enzymes revealed higher superoxide dismutase and catalase, and lower glutathione peroxidase activities in almost all Se-enriched mushrooms than in control. The obtained results suggested that Se-fortified mushrooms can cope with the accumulated cellular reactive oxygen species more successfully, and thus delay the degeneration of the culture and preserve quality for a longer period of time. Incorporation of selenium into *Coriolus versicolor* mushroom, and consequently into extract, generally enhanced its antioxidant properties. Additionally, the tested extracts expressed inhibitory effect against examined bacterial strains that have been recognized as agents of food poisoning, and therefore, they can be used as potential natural preservatives. Chemical and FTIR analyses indicated that selenium addition modified the extract composition, which mainly contained a mixture/complex of polysaccharides, proteins and polyphenols. Further work on testing the shelf life of Se-enriched mushrooms, preferably the edible ones, is required.
